# Hepatotoxicity and effectiveness of a Nevirapine-based antiretroviral therapy in HIV-infected patients with or without viral hepatitis B or C infection in Cameroon

**DOI:** 10.1186/1471-2458-10-105

**Published:** 2010-03-01

**Authors:** Jules B Tchatchueng Mbougua, Christian Laurent, Charles Kouanfack, Anke Bourgeois, Laura Ciaffi, Alexandra Calmy, Henri Gwet, Sinata Koulla-Shiro, Jacques Ducos, Eitel Mpoudi-Ngolé, Nicolas Molinari, Eric Delaporte

**Affiliations:** 1 Institut de Recherche pour le Développement, University Montpellier 1, UMR 145, Montpellier, France; 2 National advanced school of engineering, University Yaoundé 1, Yaoundé, Cameroon; 3 Central Hospital, Yaoundé, Cameroon; 4 University Hospital, Department of Infectious and Tropical Diseases, Montpellier, France; 5 Médecins Sans Frontières, Geneva, Switzerland; 6 University Hospital, Laboratory of viral hepatitis, Montpellier, France; 7 Military hospital, Yaoundé, Cameroon; 8 Department of Biostatistics, University Montpellier 1, Montpellier, France; 9 University Hospital, Department of Biostatistics, Nîmes, France

## Abstract

**Background:**

Coinfection with hepatitis B virus (HBV) or hepatitis C virus (HCV) in HIV-infected patients receiving a commonly used nevirapine-based antiretroviral therapy is a major concern for African clinicians owing to its high prevalence, the infrequent testing and treatment of viral hepatitis, and the impact of liver disease on the tolerability and effectiveness of anti-HIV treatment. We compared the hepatotoxicity and the immunological, virological and clinical effectiveness of a nevirapine-based antiretroviral therapy between patients infected with HIV only and patients coinfected with hepatitis B or C virus in Cameroon.

**Methods:**

A retrospective cohort study was conducted among HIV-1-infected patients. Plasma HBV DNA and HCV RNA were tested in positive or indeterminate samples for HBsAg or HCV antibodies, respectively. All patients received nevirapine and lamivudine plus stavudine or zidovudine.

**Results:**

Of 169 HIV-1-infected patients with a median baseline CD4 count of 135 cells/mm^3 ^(interquartile range [IQR] 67-218), 21% were coinfected with HBV or HCV. In coinfected patients, the median viral load was 2.47 × 10^7 ^IU/mL for HBV (IQR 3680-1.59 × 10^8^) and 928 000 IU/mL for HCV (IQR 178 400-2.06 × 10^6^). Multivariate analyses showed that the risk of hepatotoxicity was 2-fold higher in coinfected patients (*p *< 0.01). The response to antiretroviral therapy was however comparable between monoinfected and coinfected patients in terms of CD4 cell count increase (*p *= 0.8), HIV-1 viral load below 400 copies/mL (*p *= 0.9), death (*p *= 0.3) and death or new AIDS-defining event (*p *= 0.1). Nevirapine was replaced by a protease inhibitor in 4 patients owing to hepatotoxicity.

**Conclusion:**

This study suggests that the nevirapine-based antiretroviral therapy could be used safely as first-line treatment in patients with low CD4 cell count in Africa despite frequent coinfections with HBV or HCV and infrequent testing of these infections. Although testing for HBV and HCV should be systematically performed before initiating antiretroviral therapy, transaminases elevations at baseline or during treatment should be a decisive argument for testing when hepatitis status is unknown.

## Background

Coinfection with hepatitis B virus (HBV) or hepatitis C virus (HCV) in HIV-infected patients receiving a nevirapine-based antiretroviral therapy is a major concern for African clinicians owing to its high prevalence, the infrequent testing and treatment of viral hepatitis, and the impact of liver disease on the tolerability and effectiveness of anti-HIV treatment [1]. While HIV-related morbidity and mortality are decreasing thanks to the scaling-up of antiretroviral therapy, the impact of liver disease is likely to increase in Africa. In addition, initiation of antiretroviral therapy has been suggested in all patients coinfected with HIV and hepatitis B or C virus irrespective of the CD4 cell count.

Nevirapine, the non nucleosidic reverse transcriptase inhibitor (NNRTI) most often used in first-line treatment in combination with two nucleosidic reverse transcriptase inhibitors, is associated with early hypersensitivity reactions (generally in the first 12 weeks after treatment initiation) which can cause fulminant hepatitis leading to hepatic failure and death, and with later onset of direct drug-related hepatotoxicity leading to liver enzymes elevations [2]. The risk of hepatotoxicity is increased in patients coinfected with HBV or HCV [3-5]. The World Health Organization (WHO) therefore recommends to use nevirapine with caution and regular monitoring in patients who have baseline grade 1, 2 or 3 elevations of liver enzymes and positive or unknown HBV or HCV testing [6]. In addition, nevirapine is not recommended in patients with a grade 4 elevation of liver enzymes.

The impact of HBV or HCV coinfection on the effectiveness of a nevirapine-based antiretroviral therapy remains unclear owing to the lack of specific studies especially in the African context. A recent South African study in HBV-coinfected patients receiving the less hepatotoxic efavirenz (the second NNRTI used in first-line treatment) found similar response to antiretroviral therapy between monoinfected and coinfected patients despite higher hepatotoxicity in the latter [7]. Regardless of antiretroviral regimens (often not reported), studies on HBV or HCV coinfection in Western and Asian countries provided conflicting results with respect to CD4 cell increase, HIV suppression, AIDS progression and mortality [8-21].

We therefore compared the hepatotoxicity and the immunological, virological and clinical effectiveness of a nevirapine-based antiretroviral therapy between patients infected with HIV only and patients coinfected with HIV and hepatitis B or C virus in Cameroon [22].

## Methods

### Study design

A retrospective cohort study was conducted in two major hospitals (the Military Hospital and the Central Hospital) in Yaoundé, the capital of Cameroon, among HIV-1-infected patients enrolled from 2001 to 2003 in two clinical research projects designed to assess antiretroviral treatments [23-25]. The National Ethics Committee of Cameroon approved the study protocols, and the patients gave their written informed consent. The eligibility criteria, follow-up methods, medical and social staff, and coordinators were similar in the two projects. Briefly, patients over 18 years were eligible if they had confirmed HIV-1 infection, AIDS or a CD4 count below 350 cells/mm^3^, a Karnofsky score over 50%, and no contraindications to antiretroviral treatment, including serum liver enzyme levels less than five times the upper limit of normal (ULN) in the first project or less than three times the ULN in the second project. All patients received nevirapine and lamivudine plus stavudine or zidovudine. Tenofovir was not available. Efavirenz was temporarily substituted for nevirapine in case of concomitant tuberculosis therapy. Care (visits and laboratory exams) and drugs (antiretrovirals, and preventive and curative treatments for opportunistic infections) were provided free of charge.

### Clinical and laboratory procedures

Hepatitis B and C markers were assessed on baseline blood samples frozen at -80°C. Enzyme immunoassays (EIA) were used to detect hepatitis B surface antigens (HBsAg; Monolisa Ag HBs Plus, Bio-rad, Marnes la coquette, France) and antibodies to hepatitis C virus (anti-HCV; Ortho HCV EIA 3.0, Ortho-clinical Diagnostics, Riratan, NJ, USA). Plasma HBV DNA and HCV RNA were tested in positive or indeterminate samples for HBsAg or anti-HCV, respectively, using the Cobas Ampliprep/Cobas TaqMan quantitative assay (Roche Diagnostics GmbH, Mannheim, Germany; quantification range of 12 to 2.2 × 10^8 ^IU/mL for HBV and 15 to 6.9 × 10^7 ^IU/mL for HCV).

The CD4 cell counts were measured with a FACSCount device (Becton Dickinson, Mountain View, California, USA) at baseline and then every 6 months. The HIV-1 RNA load was determined using the Roche Amplicor HIV-1 Monitor assay (Roche Molecular Systems, Branchburg, New Jersey, USA) or the Bayer bDNA HIV-1 Quantiplex assay (Bayer Diagnostics, Emeryville, California, USA) at baseline, months 3 and 6, and then every 6 months. Serum liver enzymes (alanine aminotransferase [ALT] and aspartate aminotransferase [AST]) levels were assayed at baseline, weeks 2, 4 and 6, and then months 2, 3, 6, 9, 12, 18 and 24. Hepatotoxicity was graded with the French National Agency for Research on AIDS and Viral Hepatitis (ANRS) toxicity scale in which an ALT or AST level of 1.25-2.5 times the ULN defines grade 1, >2.5-5 times the ULN defines grade 2, >5-10 times the ULN defines grade 3 and >10 times the ULN defines grade 4 http://www.anrs.fr/content/download/2242/12805/file/ANRS-GradeEI-V1-En-2008.pdf. At each time point, hepatotoxicity grade was defined on the basis of the higher value of either ALT or AST. The HIV disease stage was determined according to the 1993 Centers for Disease Control and Prevention (CDC) classification.

### Statistical analysis

Patients were classified as coinfected if they had positive HBV DNA or HCV RNA. The analyses were based on an intention-to-treat approach. Data were censored at the time of each patient's month 24 visit or, if follow-up was shorter, at the time of the last visit or death. The χ^2 ^test and, when sample sizes were too small, Fisher's exact test were used to compare the distribution of categorical variables between the infection groups (HBV or HCV-coinfected patients *versus *HIV-monoinfected patients). For continuous variables, comparisons were based on the non parametric Mann-Whitney two-sample test.

Hepatotoxicity was first assessed by using two end points: 1) occurrence of at least one episode of grade 2 or higher hepatotoxicity, and 2) occurrence of at least one episode of grade 3 or 4 hepatotoxicity. Kaplan-Meier curves were plotted and differences between monoinfected and coinfected patients were checked for significance by the log-rank test. Cox proportional hazard models were used to compare hepatotoxicity between groups adjusted on baseline covariates. The proportional hazards assumption was evaluated by both graphic and statistical (based on Schoenfeld residuals) methods. A multivariate Poisson regression was then used to compare between groups the highest grade of hepatotoxicity reached by each patient during follow-up. The evolution of the CD4 cell count during follow-up was estimated using a mixed-effect linear regression model. Taking into account the biphasic evolution of the CD4 cell count during antiretroviral therapy [26], we allowed the slope to change after 6 months and included a quadratic effect in the second phase. In order to compare the CD4 cell counts evolution in monoinfected and coinfected patients, terms of interaction between infection status and each phase's slope or quadratic effect were tested. The CD4 cell counts were square root-transformed to approximate a normal distribution. The time to reach a viral load below 400 copies/mL, death, and death or new AIDS-defining event from treatment initiation were compared between groups by using multivariate Cox models.

Multivariate analyses were adjusted on the following baseline covariates: gender (women *versus *men), age (≥ 42 *versus *<42 years), time since diagnosis of HIV infection (<24 *versus *≥ 24 months), body mass index (≥ 21 *versus *<21 kg/m^2^), Karnofsky score (<100% *versus *100%), CDC clinical stage, CD4 cell count (≥ 100 *versus *<100 cells/mm^3^), HIV-1 viral load (<5.0 *versus *≥ 5.0 log_10 _copies/mL), hemoglobin level (≥ 10 *versus *< 10 g/dL), total lymphocytes count (≥ 1700 *versus *< 1700 cells/mm^3^), liver enzymes levels (≥ 1.25 *versus *< 1.25 × ULN), antiretroviral therapy and cotrimoxazole prophylaxis. Independent covariates associated with outcomes with a conservative *p *value of < 0.25 in univariate analysis were subsequently tested in multivariate analysis. A backward elimination procedure was used to determine the final model containing only the infection group, together with significant covariates and potential confounders. The incidence rates of hepatotoxicity, deaths, and deaths or new AIDS-defining events were expressed as the number of patients with at least one episode of the given event per 100 person-years of follow-up. For the incidence rates as for the survival analyses, data were censored at the time of the first episode of the given event. All analyses were performed using STATA 10.01 software (STATA Corporation, college Station, TX, USA).

## Results

### Patients

Of 169 HIV-1-infected patients enrolled between January 2001 and April 2003, 35 (21%) were coinfected with HBV (n = 14) or HCV (n = 21). The baseline patient's characteristics are shown in table 1. Two thirds of patients were women. Most patients were at an advanced HIV clinical stage (42% were at the CDC stage B and 44% were at stage C). The median CD4 count was 135 cells/mm^3 ^(interquartile range [IQR] 67-218). Fifteen women (13%; 12 monoinfected with HIV and 3 coinfected with HBV or HCV) had a CD4 count above 250 cells/mm^3^. No man had a CD4 count above 400 cells/mm^3^. Most characteristics were similar between HIV-monoinfected patients and those coinfected with an hepatitis virus. However, coinfected patients were older (*p *< 0.001), and had higher liver enzyme levels (*p *< 0.001). The median viral load was 2.47 × 10^7 ^IU/mL for HBV (IQR 3680-1.59 × 10^8^; range 270->2.2 × 10^8^) and 928 000 IU/mL for HCV (IQR 178 400-2.06 × 10^6^; range 640-5.5 × 10^6^) in patients coinfected with the respective virus. The median time of follow-up was 23.9 months (IQR 17.2-24.0) in monoinfected patients and 24.0 months (IQR 20.0-24.0) in coinfected patients. Half the patients received zidovudine, lamivudine and nevirapine at baseline, and the other half received stavudine, lamivudine and nevirapine. Nevirapine was interrupted during follow-up in 16 monoinfected patients (12%) and 4 coinfected patients (11%) because of tuberculosis (n = 9 and 2, respectively), adverse effects (n = 4 and 2, respectively) and antiretroviral drug resistance (n = 3 and 0, respectively).

**Table 1:** Baseline characteristics of patients by infection group

	HIV monoinfected patients (n = 134)		HBV or HCV coinfected patients (n = 35)		*p*
Women (no.)	91	(68%)	22	(63%)	0.6
Age (years)					
Median (IQR)	34.5	(28.4-39.7)	41.6	(33.7-48.8)	<0.001
<42 (no.)	113	(84%)	19	(54%)	<0.001
Time since diagnosis of HIV seropositivity (months)					
Median (IQR)	29.4	(13.5-53.4)	22.1	(8.3-43.6)	0.2
<24 (no.)	56	(42%)	18	(51%)	0.3
Body weight (Kg) [median (IQR)]	65	(55-70)	63	(54-70)	0.7
Body mass index (Kg/m2)*					
median (IQR)	23.2	(21.6-24.7)	22.2	(20.6-25.6)	0.2
≥ 21 (no.)	108	(82%)	25	(74%)	0.3
Karnofsky score >90% (no.)	72	(54%)	15	(43%)	0.3
CDC clinical stage (no.)					0.7
A	19	(14%)	4	(11%)	
B	58	(43%)	13	(37%)	
C	57	(43%)	18	(51%)	
CD4 cell count (/mm3)					
Median (IQR)	138	(67-222)	135	(68-216)	0.7
<100 (no.)	80	(60%)	22	(63%)	0.7
HIV-1 viral load (log10 copies/mL)^†^					
Median (IQR)	5.3	(4.7-5.5)	5.3	(4.8-5.7)	0.5
<5.0 (no.)	50	(37%)	11	(31%)	0.5
Hemoglobin (g/dL)					
Median (IQR)	11.0	(9.8-12.1)	11.1	(9.8-12.9)	0.6
<10 (no.)	36	(27%)	10	(29%)	0.8
Total lymphocyte count (/mm3)					
Median (IQR)	1578	(1080-2100)	1408	(1000-1900)	0.4
<1700 (no.)	77	(57%)	23	(66%)	0.4
ALT level (×ULN)					
Median (IQR)	0.6	(0.4-0.7)	0.8	(0.6-1.2)	<0.001
≥ 1.25 (no.)	8	(6%)	8	(30%)	0.002
ASAT (×ULN)^‡^					
Median (IQR)	0.8	(0.6-1.1)	1.3	(0.9-1.7)	<0.001
≥ 1.25 (no.)	21	(17%)	17	(52%)	<0.001
History of antiretroviral treatment (no.)					0.6
None	131	(98%)	34	(97%)	
HAART	1	(1%)	1	(3%)	
Nevirapine (PMTCT)	2	(2%)	0	-	
Baseline antiretroviral treatment (no.)					0.3
Zidovudine+lamivudine+nevirapine	70	(52%)	15	(43%)	
Stavudine+lamivudine+nevirapine	64	(48%)	20	(57%)	
Cotrimoxazole prophylaxis (no.)	124	(93%)	35	(100%)	0.1

ALT, alanine aminotransferase; AST, aspartate aminotransferase; ULN, upper limit of normal; PMTCT, prevention of mother-to-child transmission; HAART, highly active antiretroviral therapy

* 3 missing values; ^† ^1 missing value; ^‡ ^9 missing values

### Hepatotoxicity

A total of 1588 measures of liver enzymes were available. The number of measures per patient was similar in both infection groups (median 10, IQR 9-11, *p *= 0.2). Two patients only (both were coinfected) had a grade 2 hepatotoxicity at baseline (90 IU/L and 97 IU/L, respectively); at week 2, hepatotoxicity dropped to grade 1 in one patient and remained at grade 2 in the other patient. No patient had a baseline grade 3 or 4 hepatotoxicity.

**Figure 1:**
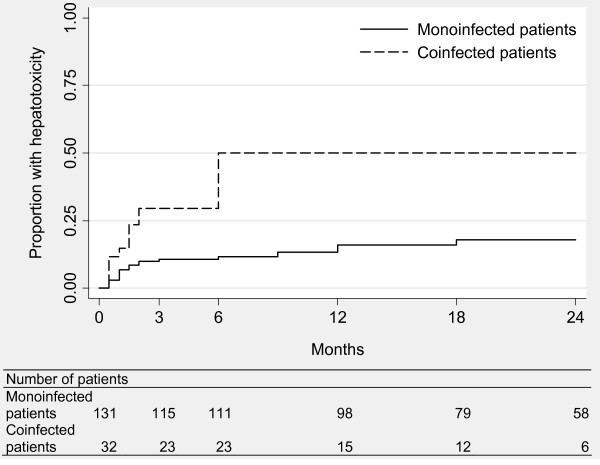
**Proportion of patients who experienced episodes of grade ≥ 2 hepatotoxicity by infection group**.

During follow-up, 39 patients experienced at least one episode of grade 2 or higher hepatotoxicity (22 monoinfected patients [16%] and 17 coinfected patients [49%]). The corresponding incidence rate was 12.4 per 100 person-years (95% confidence interval [CI] 8.2-18.9) in monoinfected patients and 54.0 per 100 person-years (CI 33.6-89.5) in coinfected patients. The Kaplan-Meier analysis showed the higher risk in coinfected patients (*p *< 0.001; figure 1). Of note, all episodes of hepatotoxicity in the coinfected patients occurred in the first six months. In monoinfected patients, such episodes occurred throughout the follow-up (although more frequently in the first months). In multivariate Cox analysis, the risk of grade ≥ 2 hepatotoxicity remained higher in coinfected patients (hazard ratio [HR] 2.94, CI 1.49-5.81, *p *= 0.002; table 2) after adjustement on age (HR 0.90, CI 0.43-1.91, *p *= 0.8), Karnofsky score (HR 0.41, CI 0.20-0.84, *p *= 0.01) and liver enzymes levels (HR 1.45, CI 0.74-2.82, *p *= 0.3). Of the 17 coinfected patients who experienced a grade ≥ 2 hepatotoxicity, 4 were positive for HBV (median viral load 1.80 × 10^8 ^IU/mL, IQR 1.04 × 10^8^-2.10 × 10^8^) and 13 were positive for HCV (median viral load 970 000 IU/mL, IQR 173 800-2.06 × 10^6^).

**Table 2:** Adjusted risks of outcomes associated with the hepatitis coinfection*

Outcome	Ratio or coefficient	95% confidence interval	*p*
Hepatotoxicity			
Grade ≥2^†^	2.94	1.49-5.81	0.002
Grade ≥3^†^	2.18	0.61-7.75	0.2
Highest grade^‡^	1.83	1.28-2.60	0.001
CD4 cell count increase^||^			
Primary slope	0.02	-0.20 to 0.24	0.9
Secondary slope^¶^	-0.04	-0.38 to 0.31	0.8
Quadratic effect^¶^	0.00	-0.00 to 0.01	0.8
Viral load < 400 copies/mL^†^	1.00	0.68-1.47	0.9
Death^†^	0.52	0.14-1.93	0.3
Death or new AIDS-defining event^†^	0.37	0.11-1.29	0.1

* Coinfected patients *versus *monoinfected patients. ^† ^Cox regression. ^‡ ^Poisson regression. ^|| ^Mixed-effect linear regression of the square root-transformed CD4 cell count. ^¶ ^From 6 months to 24 months, combined with the primary slope.

Only 12 patients (7%) experienced a grade 3 or 4 hepatotoxicity (7 monoinfected patients [5%] and 5 coinfected patients [14%]). The incidence rates were 3.6 (CI 1.7-7.6) and 9.9 per 100 person-years (CI 4.1-23.7) in monoinfected and coinfected groups, respectively. The risk tended to be higher in coinfected patients but the difference was not significant (HR 2.18, CI 0.61-7.75, *p *= 0.2; table 2) after adjustement on age (HR 1.28, CI 0.36-4.53, *p *= 0.8) and liver enzymes levels (HR 1.61, CI 0.47-5.46, *p *= 0.8). All 5 coinfected patients who experienced a grade 3 or 4 hepatotoxicity were HCV positive; their median viral load was 970 000 IU/mL (IQR 320 000-1.22 × 10^6^).

The higher risk for hepatotoxicity in coinfected patients was confirmed by the multivariate Poisson regression analysis (incidence risk ratio [IRR] 1.83, CI 1.28-2.60, *p *= 0.001; table 2) after adjustement on age (IRR 0.83, CI 0.56-1.24, *p *= 0.4) and Karnofsky score (IRR 0.54, CI 0.39-0.76, *p *< 0.001).

Three patients developed a rash in the first four weeks (two women with baseline CD4 counts of 31 and 307 cells/mm^3^, respectively, and one man with a baseline CD4 count of 18 cells/mm^3^). Only the male patient was coinfected (HBV viral load 4.88 × 10^7 ^IU/mL). Nevirapine was replaced by a protease inhibitor in all three patients. The woman with a high CD4 cell count experienced simultaneously a grade 3 hepatotoxicity. Nevirapine was also replaced by a protease inhibitor in one other (monoinfected) patient with an isolated grade 3 hepatotoxicity. No patient had clinical hepatitis.

### Immunological response

**Figure 2:**
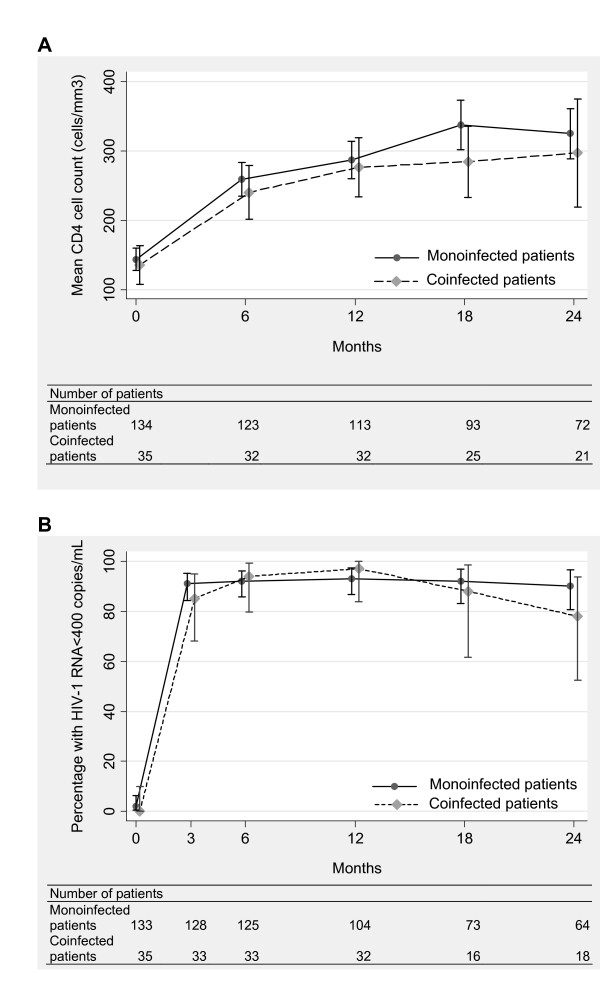
**Immunological and virological responses to antiretroviral therapy by infection group: mean CD4 cell count (A), and percentage of patients with plasma HIV-1 viral load below 400 copies/mL (B)**. Bars indicate 95% confidence intervals.

The number of CD4 cell counts per patient was comparable between the monoinfected group (median 5, IQR 3-5) and the coinfected group (median 4, IQR 4-5; *p *= 0.5). As shown in figure 2A, the CD4 cell count increased after treatment initiation from 143 to 325 cells/mm^3 ^in monoinfected patients and from 136 to 297 cells/mm^3 ^in coinfected patients. The square root CD4 cell count increase was not significantly different between monoinfected and coinfected patients (either in the first 6 months [*p *= 0.9] and thereafter [*p *= 0.8]; table 2) after adjustement on gender (coefficient 1.59, CI 0.41 to 2.77, *p *= 0.008), age (coefficient -1.07, CI -2.43 to 0.29, *p *= 0.1), HIV-1 viral load (coefficient 1.55, CI 0.40 to 2.70, *p *= 0.008), hemoglobin level (coefficient 2.43, CI 1.18 to 3.67, *p *< 0.001) and total lymphocyte count (coefficient 2.93, CI 1.83 to 4.03, *p *< 0.001).

### Virological response

The number of HIV-1 RNA measures per patient was similar in both infection groups (median 5, IQR 4-6, *p *= 0.8). The proportion of patients with viral load below 400 copies/mL was 91% and 85% after 3 months and 91% and 78% after 24 months in monoinfected and coinfected patients, respectively (figure 2B). The time for reaching a viral load below 400 copies/mL did not differ between the groups (HR 1.00, CI 0.68-1.47, *p *= 0.9; table 2). No other variable remained in the model.

### Deaths or new AIDS-defining events

**Figure 3:**
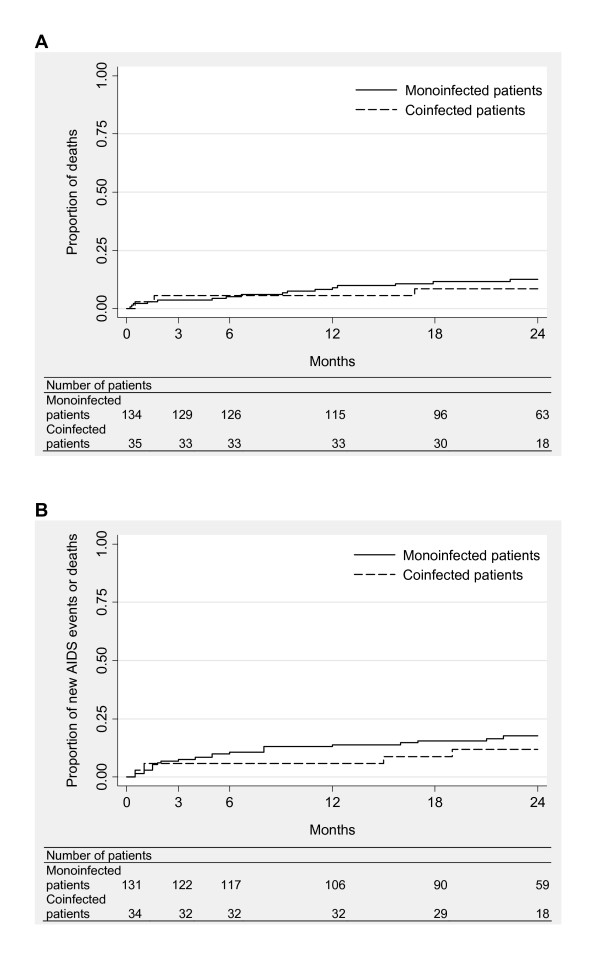
**Clinical progression by infection group: progression to death (A), and progression to death or new AIDS-defining event (B)**.

There were 16 deaths (12%) in monoinfected patients and 3 (9%) in those coinfected. The mortality rates were 7.2 (CI 4.4-11.7) and 4.8 (CI 1.4-15.0) per 100 person-years, respectively. The Kaplan-Meier curves did not differ significantly between the groups (*p *= 0.6, figure 3A). In multivariate analysis, death was not associated with coinfection (HR 0.52, CI 0.14-1.93, *p *= 0.3; table 2) after adjustement on gender (HR 0.29, CI 0.11-0.77, *p *= 0.01), age (HR 1.90, CI 0.68-5.29, *p *= 0.2), CD4 cell count (HR 0.15, CI 0.04-0.55, *p *= 0.004) and hemoglobin level (HR 0.28, CI 0.11-0.74, *p *= 0.01). In the monoinfected group, the deaths were related to advanced HIV disease (n = 5), poor general health (n = 5), multifocal tuberculosis, pulmonary infection, wasting, malaria, pancreatitis and hepatic carcinoma (n = 1 each). In the coinfected group, death was related to advanced HIV disease, poor general health and persistent fever of unknown origin (n = 1 each); the first two patients had taken traditional medicines.

Deaths or new AIDS-defining events occurred in 25 monoinfected patients (19%) and 5 coinfected patients (14%). The respective incidence rates were 10.6 (CI 7.0-16.1) and 6.7 per 100 person-years (CI 2.5-17.8). The Kaplan-Meier curves showed no significant difference between the groups (*p *= 0.5, figure 3B). In multivariate analysis, the occurrence of deaths or new AIDS-defining events was not associated with coinfection (HR 0.37, CI 0.11-1.29, *p *= 0.1; table 2) after adjustement on age (HR 1.33, CI 0.53-3.32, *p *= 0.5) and body mass index (HR 4.40, CI 1.98-9.78, *p *< 0.001).

## Discussion

This study in Cameroon showed a higher hepatotoxicity in HBV or HCV-coinfected patients receiving a nevirapine-based antiretroviral therapy than in their HIV-monoinfected homologues. However, this adverse event did not impact negatively on the effectiveness of treatment.

Altogether, our analyses showed that the risk of hepatotoxicity was 2-fold higher in hepatitis B or C coinfected patients. This finding is consistent with previous reports [11,17,27,28], especially with a study in patients receiving a nevirapine-based treatment in United States [29]. Of note, HCV-coinfected patients accounted for 76% of overall coinfected patients with liver enzymes elevations >2.5 times the ULN (ALT and/or AST levels) during treatment and 100% of those with liver enzymes elevations >5 times the ULN (as compared with 60% of the study's coinfected group). It should however be noted that HCV infection was not treated while HBV infection was treated with lamivudine monotherapy. Although anti-HBV lamivudine monotherapy has been shown to lead to frequent emergence of drug resistance [30] and, consequently, to possible acute hepatitis, fulminant hepatic failure, and death [3], lamivudine was likely to be effective against HBV in the first months of treatment when most episodes of hepatotoxicity occurred. Importantly, up to half the coinfected patients experienced liver enzymes elevations >2.5 times the ULN.

Overall, the hepatic tolerability of the nevirapine-based antiretroviral therapy was as expected. Seven percents of patients experienced liver enzymes elevations >5 times the ULN. In a comprehensive analysis of 17 randomized clinical trials of nevirapine, the rate was 10% after one year of treatment [31]. A higher rate of 17% has been found in South African patients who had a mean baseline CD4 count of 398 cells/mm^3 ^[32]. Our lower rate should be explained by the profound immunodeficiency of our patients at the time of treatment initiation (median CD4 count of 135 cells/mm^3^). Indeed, transaminases elevations >5 times the ULN in patients receiving nevirapine has been associated with sex-dependant CD4 cell count [5]. Thus, nevirapine should be avoided in women with a CD4 count above 250 cells/mm^3 ^and in men with a CD4 count above 400 cells/mm^3^. In our study, only 13% of women and no man had a CD4 cell count above these respective cuttoffs. Most transaminases elevations in our patients were asymptomatic. Only 2% of patients had a rash following treatment initiation. In their analysis, Dieterich *et al *found a rash in 2.2% of patients [31]. On the other hand, the incidence rates of liver enzymes elevations >5 times the ULN in our monoinfected and coinfected patients were comparable to those reported in South African patients receiving an efavirenz-based antiretroviral therapy [7].

The immunological response to the nevirapine-based antiretroviral therapy was comparable between coinfected and monoinfected patients, in accordance with several studies irrespective of the treatment prescribed [7,11,13,14,18,20]. In contrast, other studies found delayed CD4 cell count recovery in coinfected patients especially in those coinfected with HCV [8-10,21]. However, this lower immunological response was not sustained beyond the first few months in the Thai and Swiss studies [10,33].

The virological response also was comparable between patients coinfected with an hepatitis virus and patients infected with HIV only. Increased risk for virological failure has been reported in HBV-coinfected patients in Taiwan [11]. However, most other studies like ours did not find any difference [7,9,10,17,21].

The clinical progression did not differ significantly between coinfected and monoinfected patients but treatment was initiated late (median CD4 count of 135 cells/mm^3^). Surprisingly, deaths or new AIDS-defining events were less common in coinfected patients. A possible explanation could be a close medical supervision of coinfected patients owing to frequent liver enzymes elevations. Higher rates of deaths (from any cause, liver-related causes and/or AIDS-related causes) and/or new AIDS-defining events in patients coinfected with HBV or HCV has been reported by some [8,11-16,18,20] but not all studies [7,9,10,17]. In our study, only one death was from a liver-related cause and this was recorded in a monoinfected patient. Intake of traditional medicines which have a potential for hepatic toxicity was however reported in two of the three coinfected patients who died.

The main limitation to our study was the relatively small sample size. However, a significant higher risk of hepatotoxicity in coinfected patients was demonstrated. Also, the absence of viral hepatitis impact on immunological and virological responses to antiretroviral therapy was strongly suggested by the regression coefficients and hazard ratio, respectively. Finally, the risk of clinical progression tended to decrease in coinfected patients which is likely to be related to an unmeasured confounding factor. The exclusion of patients presenting with serum liver enzyme levels higher than three or five times the ULN values (depending on the initial study) could have led to a selection bias (exclusion of patients with an advanced hepatitis clinical stage) but this is concordant with the recommendations for nevirapine use. Classification biases for hepatitis status could also not be ruled out because seronegative blood samples were not tested for HBV DNA or HCV RNA. Higher rates of negative HBsAg or anti-HCV results in viraemic samples have been observed in immunocompromised HIV-infected patients [3,4]. In addition, unrepeated testing of HBV and HCV during follow-up did not allow to identify new or reactivated hepatitis infections. New infections, if any, were however likely to be rare owing to the study's duration and, for HBV, the usual childhood infections in Africa through close contacts within households and, to a lesser extent, vertical transmission (unclear modes of transmission for HCV) [1]. On the other hand, no symptomatic reactivated hepatitis infections were recorded. In contrast, classification of patients on the basis of HBV DNA and HCV RNA is clearly a strength of our study while most other studies only used serological assays.

## Conclusion

This study suggests that the nevirapine-based antiretroviral therapy could be used safely as first-line treatment in patients with low CD4 cell count in Africa despite frequent coinfections with HBV or HCV and infrequent testing of these infections. Although testing for HBV and HCV should be systematically performed before initiating antiretroviral therapy, transaminases elevations at baseline or during treatment should be a decisive argument for testing when hepatitis status is unknown.

## Competing interests

The authors declare that they have no competing interests.

## Authors' contributions

JBTM analysed the data and, with CL, wrote the first draft of the report. CL also designed the study and contributed to data collection, analysis and interpretation of data. HG and NM contributed to data analysis. CK, AB, LC, AC, SKS, JD, EMN and ED contributed to data collection, interpretation of data and drafting of manuscript. All authors read and approved the final draft of the manuscript.

## Pre-publication history

The pre-publication history for this paper can be accessed here:

http://www.biomedcentral.com/1471-2458/10/105/prepub
